# Solvent-free fabrication of thermally conductive insulating epoxy composites with boron nitride nanoplatelets as fillers

**DOI:** 10.1186/1556-276X-9-643

**Published:** 2014-11-29

**Authors:** Zifeng Wang, Yuqiao Fu, Wenjun Meng, Chunyi Zhi

**Affiliations:** 1 Department of Physics and Materials Science, City University of Hong Kong, 83 Tat Chee Avenue, Kowloon, Hong Kong, People’s Republic of China; 2 Shenzhen Research Institute, City University of Hong Kong, Shenzhen, People’s Republic of China

**Keywords:** Boron nitride nanoplatelets, Thermal conductivity, Epoxy composites, Solvent-free fabrication

## Abstract

A solvent-free method for the fabrication of thermally conductive epoxy-boron nitride (BN) nanoplatelet composite material is developed in this study. By this method, polymer composites with nearly any filler fractions can be easily fabricated. The maximum thermal conductivity reaches 5.24 W/mK, which is 1,600% improvement in comparison with that of pristine epoxy material. In addition, the as-fabricated samples exhibit excellent overall performances with great mechanical property and thermal stability well preserved.

## Background

With the miniaturization and integration trend of transistors in the microelectronic devices, heat management has become an important issue in manufacturing more powerful and reliable devices [1,2]. In order to realize this purpose, thermally conductive but electrically insulating materials are selected as thermal interface materials for heat dissipation. Polymer-based composite materials with inorganic fillers incorporated are potential candidates and have attracted increasing attention for their processability and low density [3-7]. As is known to all, commonly used polymers, such as epoxy resin, polyacrylic resin, and polyurethane have a thermal conductivity in the range of 0.1 to 0.4 W/mK, which is far below the required levels. Therefore, to improve thermal conductivity, high fraction of inorganic fillers should be added into the matrix [8,9]. Kim et al. reported a thermal conductivity of 2.85 W/mK of epoxy-boron nitride (BN)-filled composite at a filler fraction of 70 wt.% [10]. Huang et al. reported a maximum thermal conductivity around 6 W/mK with a KBM303-treated AlN particles at a filler fraction of 65 vol.% [11]. Other types of fillers like Al_2_O_3_, with a thermal conductivity of 4.3 W/mK at a filler fraction of 60 vol.%, have also been reported [12].

In spite of the progresses achieved, however, problems still exist. In order to disperse high loading fraction of fillers in the matrix, toxic organic solvents, such as dimethylformamide (DMF), tetrahydrofuran (THF), and methyl ethyl ketone (MEK), are sometimes inevitable for composite materials’ fabrication, which is not environmentally friendly enough [7,11,13-15]. In addition, effective removal of solvents remains a difficulty when bulk sample instead of films is fabricated. These problems are especially stubborn for fabrication of epoxy-based composite materials.

Boron nitride (BN) with a band gap of 6.0 eV possesses a high intrinsic in-plane thermal conductivity of 30 to 300 W/mK and the theoretical thermal conductivity of a BN nanotube may even high up to approximately 3,000 W/mK [12,16,17]. By virtue of its unique thermal conductivity, BN has been used intensively as ideal filler for thermally conductive composite materials. In terms of BN-filled polymer composite, appropriate highly effective mixing methods are necessary to produce material with high thermal conductivity [18,19]. On the one hand, it was found that the thermal conductivity of polyethylene-graphite composite materials with different mixing methods can be ranked as: powder mixing > solution mixing > roll-mill mixing > melt mixing [20]. On the other hand, Jonathan et al. demonstrated that mechanical shear is a facile and effective method to exfoliate materials with layered structure [21]. Therefore, it is believed that the combination of these two methods, i.e. utilizing powder mixing mechanically exfoliated BN nanoplatelets (BNNPs), is a promising approach for fabricating highly thermally conductive polymeric composite filled with exfoliated BNNPs.

In this article, we present a facile solvent-free method for fabrication of BNNPs/epoxy resin composite materials. By utilizing the solid-state epoxy resin, the composite can be fabricated at almost any BNNP loading fraction without the help of organic solvent. The morphology, thermal conductivity, mechanical property, and density of the fabricated composite materials are studied systematically.

## Methods

### Materials

Solid-state epoxy resin (E13) and hardener (TP41) were purchased from TECH-POWER (HUANGSHAN) LTD, China. Raw BN powder was purchased from Zibo Jonye Ceramic Technologies Co., Ltd, Shandong province, China. KH550 silane coupling agent was purchased from Wancheng Chemical, China. All chemicals and materials were used as received without further purification. In order to form bulk-sized samples, a properly designed mold was used in the fabrication.

### Silane treatment for BNNPs

BN powder was treated with silane coupling agent (KH550) following the method reported by Chung et al. [22]. A silane-water solution was made at selected concentration in a flask. Then, BN powder was weighed at selected weight (BN: silane = 100:2.4) and added into the solution under magnetic stirring. After that, the flask was heated up to 65°C in a water bath with continued magnetic stirring for about 30 min. Finally, the treated powder was rinsed with water by filtration and the collected powder was dried in an oven at 110°C for 12 h.

### Epoxy resin-BNNP composite fabrication

**Figure 1:**
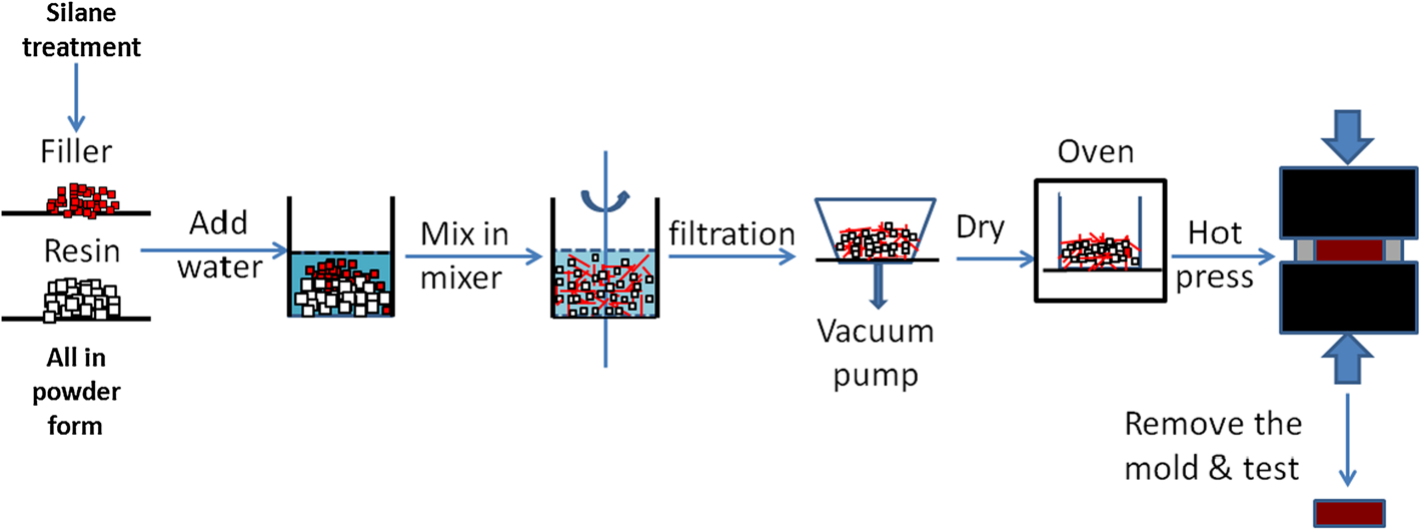
Developed solvent-free fabrication method for epoxy-BNNPs composite materials.

A series of epoxy resin-BNNP composites were fabricated with selected filler loading fraction from 0 to 70 wt.%, with a 10 wt.% increment for each. Epoxy resin powder (E13) and curing agent (TP41) were weighed at a weight ratio of 5:2. Then, epoxy resin and hardener as well as BNNPs, which has been dried for 12 h, were put into a mixer and 400 ml deionized water was added. Then, the mixture was mixed in the water for 10 min by strong stirring, followed by filtration. The collected blend powder was dried in the oven at 50°C for 8 h.The as-prepared blend powder was then put into the mold that was pre-designed for the fabrication. In order that the sample can be easily removed from the mold, mold release agent and two pieces of thin Teflon paper were used. The mold with powder filled in was put into hydraulic press made by DAKE and heated up to 180°C for 1 h for the curing process. Then, hot press was conducted with a pressure of 40 MPa at 100°C for 30 min. Finally, the sample as well as the mold was cooled down to room temperature in air. The general procedure of the fabrication is showed in Figure 1.

### Characterization and measurements

The morphology and structural investigation were performed by scanning electron microscopy (SEM, JSM6335F FEG and JEOL JSM-820, Akishima-shi, Tokyo, Japan) and transmission electron microscopy (TEM, CM20). Thermal conductivities of as-fabricated samples were measured by DZDR-S thermal conductivity tester, which utilizes the transient plane source method. A plane sensor was sandwiched by two pieces of specimens with flat surface and thermal conductivity on the vertical direction perpendicular to the testing plane can be read from the screen directly. The density and hardness of the material were measured successively, by electronic densimeter (MD-300S) and Vickers hardness tester (FV-700), respectively.

## Results and discussion

**Figure 2:**
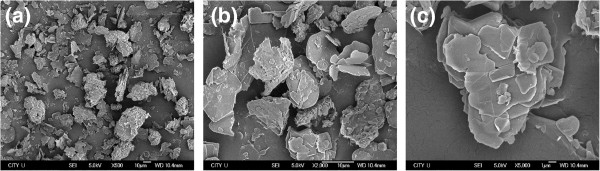
**Typical SEM images of as-purchased BN powder at different magnifications. (a)** BN powder at 500 magnification; **(b)** BN powder at 2,000 magnification; **(c)** BN powder at 5,000 magnification.

**Figure 3:**
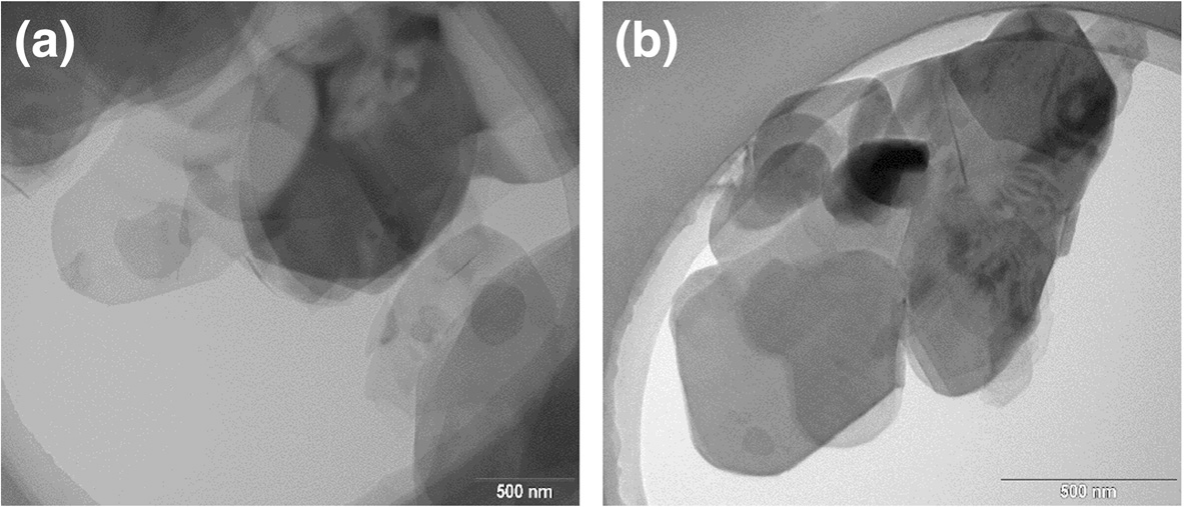
Typical TEM images of BNNPs after exfoliation by mixer (a and b).

Figure 2 shows the representative SEM images of the pristine BNNPs, of which the average size is around 10 μm. Clear platelet-like structure was demonstrated. The large size particles can be identified to be assembly structure of platelets, which may enable an easy exfoliation with shearing force. Figure 3 shows the representative TEM images of BNNPs after exfoliation by the mixer for 10 min. According to the images, the exfoliated BNNPs show a high aspect ratio, with lateral size in micron and average thickness around tens of nanometers. This is favorable for forming thermally conductive paths in the matrix. As is known to all, thermal conductivity increases rapidly after reaching the so-called percolation threshold, at which continuously conductive path is formed. Also, fillers with larger average sizes have the advantage for obtaining maximum packing density easier [23]. Apart from that, referring to Nielsen’s model in the prediction of thermal conductivity, geometry of filler or aspect ratio of the filler plays an important role for the dramatic improvement of thermal conductivity of the composite at high loading fraction compared with fillers with low aspect ratios [24].

**Figure 4:**
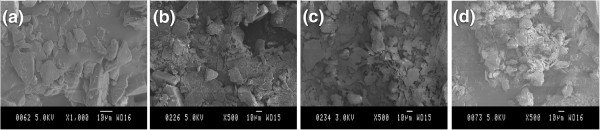
**SEM images of typical blend powders. (a)** SEM images of pure epoxy powder; **(b)** SEM images of 10 wt.% BNNP-epoxy powder; **(c)** SEM images of 40 wt.% BNNP-epoxy powder; **(d)** SEM images of 70 wt.% BNNP-epoxy powder.

**Figure 5:**
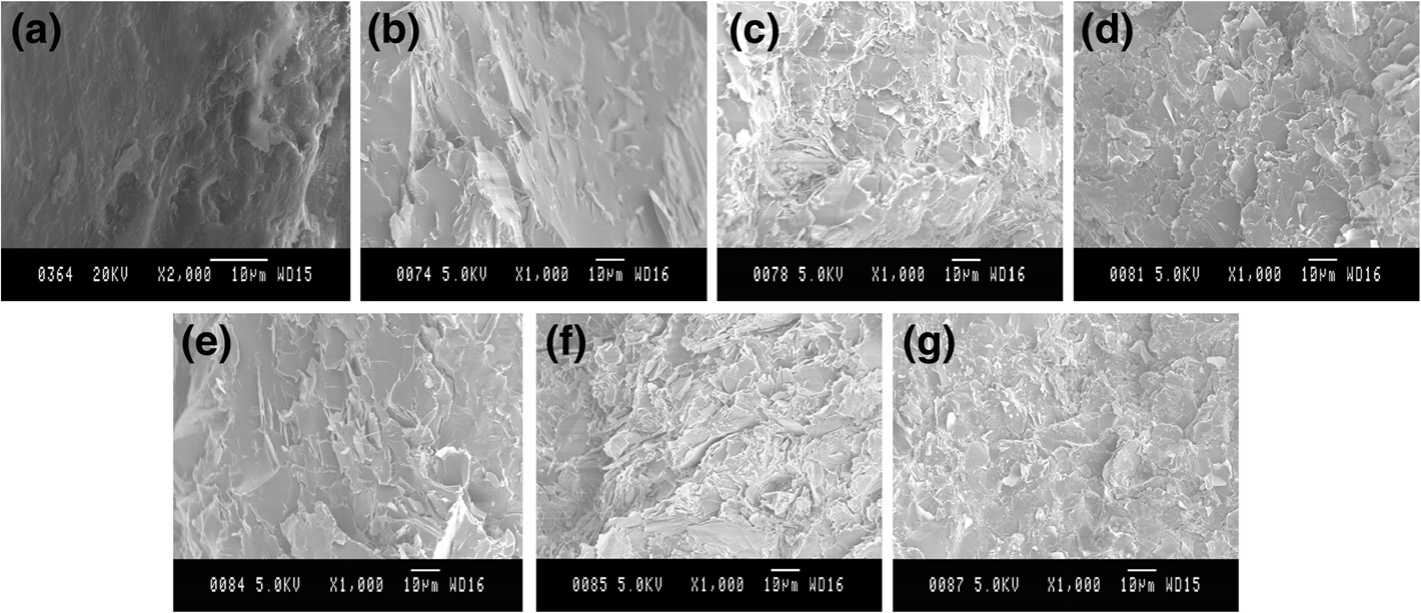
**SEM fractured surface images of typical silane-treated BNNP-epoxy samples and non-treated samples. (a)** SEM image of pure epoxy resin; **(b to d)** SEM images of non-silane-treated BNNP-epoxy composites with BNNP loading fractions of 10, 40, and 70 wt.%, respectively; **(e to g)** SEM images of silane-treated BNNP-epoxy composites with BN loading fractions of 10, 40, and 70 wt.%, respectively.

**Figure 6:**
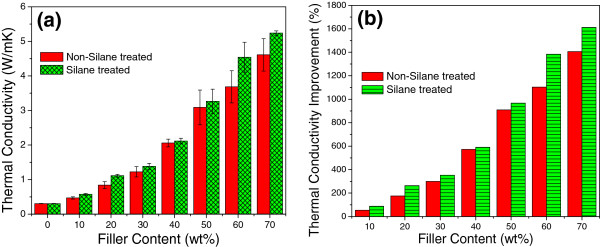
**Thermal conductivity and improvement of as-made epoxy-BNNP composites. (a)** Thermal conductivity of silane-treated BNNP and non-silane-treated BNNP-epoxy composite materials; **(b)** thermal conductivity improvement (κcomposites/κepoxy) of the two types of composite materials compared with pure epoxy.

**Figure 7:**
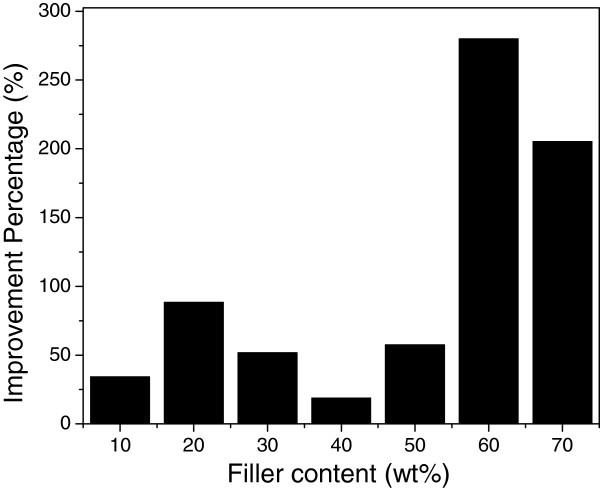
**Thermal conductivity improvements at each of the filler loading.** This is defined as improvement percentage of the thermal conductivity of silane-treated one - improvement percentage of the non-treated one at each of the corresponding filler content in Figure 6a.

Figure 4 shows the typical SEM images of as-fabricated blend powder at filler fraction of 0, 10, 40, and 70 wt.%, respectively. BNNPs remain platelet-like shape and become much thinner than pristine BN particles due to shearing force exfoliation. They are uniformly dispersed in the epoxy resin powder, which proves the effectiveness of mixing method even without the help of organic solvent. The powder epoxy resin will soften and become liquid with low viscosity during the curing process, which will result in the infiltration of the BNNPs. Zhu et al. discussed the interaction between epoxy matrix and ceramics filler with regarding to the viscosity of the composite system [25]. The addition of BN filler will cause significant increase in viscosity which confines the chain mobility of epoxy and increase the difficulties in degasing and solvent removal. In our current study, however, this problem can be partially circumvented by virtue of non-solvent system and is promising for the fabrication of epoxy-BNNP composite material at high filler fractions.Silane coupling agent is effective in modifying the interface adhesion in our study and was used to achieve better dispersion of BNNPs and more effective improvement of thermal conductivity of the fabricated composites. Hereafter, samples without silane treatment are denoted as EP/BN_10 to EP/BN_70 with ten increments each time while samples with silane treatment are denoted as EP/BN_10(S) to EP/BN_70(S), respectively. The representative SEM images of as-made composite materials are presented in Figure 5. Pure epoxy, EP/BN_10, EP/BN_40, EP/BN_70, EP/BN_10(S), EP/BN_40(S), and EP/BN_70(S) are selected for investigations and comparisons.As shown in SEM images of the fractured surfaces, the BNNPs are well dispersed and randomly oriented in the epoxy matrix. Platelet-like shape of BN is clearly revealed. Upon surface treatment, there is no significant difference which can be observed under SEM observation. At high fractions, BNNP loaded samples, such as in samples of EP/BN_40 or EP/BN_40(S) and EP/BN_70 or EP/BN_70(S), as shown in Figure 5c, d, f, g, continuous thermally conductive paths can be considered to be formed as individual BNNPs start to overlap with each other, which is essentially important for effective heat conduction. Apart from that, composites with non-silane-treated BNNPs may contain more voids than the composites with silane-treated BNNPs, as indicated in Figure 5c, f, as well as 5d, g. This may indicate a better interfacial adhesion between silane-treated BNNPs and epoxy matrix.Figure 6 presents thermal conductivity values and thermal conductivity improvements (thermal conductivity ratio between composite and pristine epoxy) of as-fabricated composites. From Figure 6a, it can be clearly seen that the thermal conductivity of composite materials increases remarkably with increased filler loading fraction, for both silane-treated and non-silane-treated samples. The highest thermal conductivity value obtained by non-silane-treated sample is about 4.61 W/mK at 70 wt.% loading fraction while the highest thermal conductivity value obtained by silane-treated sample is high up to 5.24 W/mK. Differences in thermal conductivity improvement of the two series of samples are also observable compared with pure epoxy with a thermal conductivity value of 0.31 W/mK. At both 70 wt.% BNNP fraction, the improvement for non-silane-treated sample EP/BN_70 is approximately 1,400% while that is approximately 1,600% for silane-treated sample EP/BN_70(S), as shown in Figure 6b.Another feature observed from Figure 6 is that the enhancement values of thermal conductivity is much less effective at low filler fractions, especially from 10 to 30 wt.%, than that at high filler fractions, for instance, from 50 to 70 wt.%, which is consistent with the percolation theory. There is only 0.76 W/mK thermal conductivity increase obtained (from 0.47 to 1.22 W/mK) when the filler fraction increase from 10 to 30 wt.% for the non-silane-treated samples. The improvement ratio is about 246%. At high filler fractions, e.g. from samples EP/BN_50 to EP/BN_70, the increase of thermal conductivity is high up to 1.52 W/mK and the improvement is more than 490%. This is because at low filler fraction, long-range thermally conductive paths may not steadily form to reach the percolation threshold. However, it can be assumed that at these high filler fractions, continuous relatively long-range thermally conductive paths are formed and reach the percolation threshold so that a relatively rapid thermal conductivity increase is witnessed.The function of silane treatment is also reflected on the differences in the thermal conductivity between non-silane-treated and silane-treated BNNPs. Distinguishable improvement of thermal conductivity is witnessed at high filler fractions rather than lower ones. As indicated in Figure 7, silane treatment improvement of the thermal conductivity at each of the filler loading fraction is more effective at high filler fraction. For example, at 60 and 70 wt.% BNNP fractions, more than 200% improvement is achieved, while at low filler fractions of 10 to 50 wt.%, the improvements are normally less than 100%.

Ignoring the engineering and fabrication processes, the mechanisms of thermal conductivity improvement can be further phenomenologically discussed. At low filler fractions, long-range thermally conductive paths are not steadily formed, i.e. BNNPs are not effectively contacted with each other. As a result of silane treatment, although filler dispersion might be improved, thermal resistance actually exists at the interfaces between filler and matrix, which causes serious phonon scattering and deficiency. At high filler fractions, however, long-range thermally conductive paths are formed as there are enough BNNPs involved in the heat conduction. In this case, agglomeration of highly concentrated fillers and dispersion of them in the matrix become the other problems. These problems can be partially overcome by silane treatment to BNNPs. As is mentioned above, silane coupling agent can enhance interfacial adhesion between polymer matrixes and inorganic fillers thus improving the dispersion and preventing serious agglomeration of the fillers. Although interfacial thermal resistance still exists, which may cause deficiency in heat conduction, the improvement of thermal conductivity is greater due to the suppressed phonon scattering at interfaces. In our study, silane treatment works most effectively at 60% BNNP fraction for the thermal conductivity improvement.

**Figure 8:**
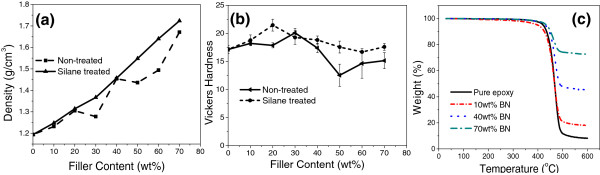
**Density, Vickers hardness, and TGA measurements of as-made samples. (a)** Densities of samples with silane-treated and non-silane-treated BNNP fillers, respectively; **(b)** Vickers hardness of samples with silane-treated and non-silane-treated BNNP fillers; **(c)** TGA measurement of 0, 10, 40, and 70 wt.% BNNP-epoxy composite materials.

Except the thermal conductivities, the density, Vickers hardness as a representative of mechanical properties and thermogravimetry analysis (TGA) of the fabricated composite materials are studied as well to reveal the overall performances of them. The results are showed in Figure 8. Figure 8a shows the density of silane-treated and non-silane-treated BNNP/epoxy composites fabricated. In general, higher density is obtained for those with higher filler fractions, which is due to the addition of BNNPs which has higher density than epoxy. There is a nearly linear increase for the density of the composites with silane-treated BNNPs as fillers. However, the composite samples with non-silane-treated BNNPs as fillers present a fluctuated pattern. This phenomenon indicates a higher sample quality for the silane-treated BNNP/epoxy composites than the non-silane-treated ones. In addition, thermal conductivity *k* is usually given by the equation shown as k = αρ*C*_*p*_, where *α* is the thermal diffusivity, *ρ* is the density, and C_*p*_ is the specific heat capacity. Therefore, it can be concluded that the observed enhancements of thermal conductivities are contributed by not only the high thermal diffusivity of BNNPs but also higher density of the composite material.Even at very high filler fraction, the Vickers hardness of the fabricated composite materials can be maintained very well, as shown in Figure 8b. The pristine epoxy material has a hardness of approximately 17, while even for samples of EP/BN_70 and EP/BN_70(S), the hardness is kept at approximately 15. Moreover, the Vickers hardness of silane-treated samples is generally higher than those without silane treatment according to Figure 8b, indicating better sample quality, which is consistent with the density data shown in Figure 8a. The overall pattern of hardness of the samples fluctuates with a large drop of hardness witnessed for EP/BN_50 which indicates efforts are still needed to improve sample quality.

Thermogravimetric analyses (TGAs) are conducted in nitrogen (N_2_) for pure epoxy, EP/BN_10, EP/BN_40, and EP/BN_70, as examples. As shown in Figure 8c, the thermal decomposition temperature of the four reflected by TGA spectra keeps almost the same at around 450°C. This implies that the addition of BNNPs does not change the decomposition temperature of the epoxy resin matrix. The epoxy resin used in our studies shows a thermal stability in nitrogen up to nearly 350°C without significant weight loss, which reveals an excellent thermal stability of the fabricated composite materials.

## Conclusions

A facile solvent-free process is developed to fabricate thermally conductive electrically insulating epoxy-BNNP composites with various filler fractions. Benefiting from solid-state epoxy resin adopted, the epoxy resin and BNNPs are mixed uniformly only by mechanical mixing with a juice maker. Without the very difficult desolvation processes, the developed method can be used to fabricate bulk-sized composite samples with very high filler fractions. The fabricated epoxy-BNNPs exhibit steadily improved thermal conductivity up to 5.24 W/mK at 70 wt.% filler fraction, which is 1,600% better than that of pristine epoxy material. Surface treatment of BNNPs by silane coupling agent is demonstrated to be effective for further enhancement of the thermal conductivity of the composites, especially at high filler fractions. In addition, the as-fabricated composite materials exhibit excellent overall performance with high density, well preserved hardness, and great thermal stability. Further improvements of the fabrication method in the future can be realized in these aspects, such as: (1) modify the method for non-solid state material; (2) combine chemical modifications with our method to obtain better thermal conductivity values; (3) extend time of mixing to obtain a better dispersion. With those optimizations, the fabricated epoxy-BNNP composite materials and developed facile solvent-free fabrication method are promising for various heat dissipation-oriented applications.

## Competing interests

The authors declare that they have no competing interests.

## Authors’ contributions

The work presented above was completed by the collaboration of all the authors mentioned. ZFW analyzed all the data and completed writing the paper. YQF and WJM purchased all the needed chemicals and raw materials and participated in the experimental section. All authors read and approved the final manuscripts.
